# The Role of Citrate Homeostasis in Merkel Cell Carcinoma Pathogenesis

**DOI:** 10.3390/cancers14143425

**Published:** 2022-07-14

**Authors:** Konstantin Drexler, Barbara Schwertner, Silke Haerteis, Thiha Aung, Mark Berneburg, Edward K. Geissler, Maria E. Mycielska, Sebastian Haferkamp

**Affiliations:** 1 Department of Dermatology, University Medical Center, 93053 Regensburg, Germany; barbara.schwertner@ukr.de (B.S.); mark.berneburg@ukr.de (M.B.); sebastian.haferkamp@ukr.de (S.H.); 2 Institute for Molecular and Cellular Anatomy, University of Regensburg, 93053 Regensburg, Germany; silke.haerteis@vkl.uni-regensburg.de (S.H.); thiha.aung@vkl.uni-regensburg.de (T.A.); 3 Faculty of Applied Healthcare Science, Deggendorf Institute of Technology, 94469 Deggendorf, Germany; 4 Department of Surgery, Section of Experimental Surgery, University Hospital Regensburg, 93053 Regensburg, Germany; edward.geissler@klinik.uni-regensburg.de; 5 Department of Structural Biology, Institute of Biophysics and Physical Biochemistry, University of Regensburg, Universitätsstrasse 31, 93053 Regensburg, Germany; maria.mycielska@biologie.uni-regensburg.de

**Keywords:** cancer, Merkel cell carcinoma, citrate, pmCiC, gluconate

## Abstract

**Simple Summary:**

Merkel cell carcinoma (MCC) is a rare but highly aggressive skin cancer. Despite important progress, overall understanding of the events that drive MCC carcinogenesis remains incomplete. We discovered that the plasma membrane citrate transporter (pmCiC) is upregulated in Merkel cell carcinoma cell lines. Cancer cells import extracellular citrate via pmCiC to support their metabolism, which is critical to support proliferation and metastatic spread. In this study, we show that inhibition of pmCiC can decrease the growth rate of Merkel cell carcinoma cell lines. Targeting pmCiC and thereby the tumor metabolism should be considered further as a potential anti-cancer therapy.

**Abstract:**

Merkel cell carcinoma (MCC) is a rare but highly aggressive tumor of the skin with a poor prognosis. The factors driving this cancer must be better understood in order to discover novel targets for more effective therapies. In the search for targets, we followed our interest in citrate as a central and critical metabolite linked to fatty acid synthesis in cancer development. A key to citrate uptake in cancer cells is the high expression of the plasma membrane citrate transporter (pmCiC), which is upregulated in the different adenocarcinoma types tested so far. In this study, we show that the pmCiC is also highly expressed in Merkel cell carcinoma cell lines by western blot and human tissues by immunohistochemistry staining. In the presence of extracellular citrate, MCC cells show an increased proliferation rate in vitro; a specific pmCiC inhibitor (Na^+^-gluconate) blocks this citrate-induced proliferation. Furthermore, the 3D in vivo Chick Chorioallantoic Membrane (CAM) model showed that the application of Na^+^-gluconate also decreases Merkel cell carcinoma growth. Based on our results, we conclude that pmCiC and extracellular citrate uptake should be considered further as a potential novel target for the treatment of Merkel cell carcinoma.

## 1. Introduction

Merkel cell carcinoma (MCC) is an aggressive tumor of the skin with a rising incidence [1] that predominately affects elderly Caucasians (median age at diagnosis is 75–80). Primary tumors have a predilection for sun exposed areas of the skin [2,3]. In addition to UV-radiation, immune deficiencies are important risk factors for the development of MCCs [3]; the risk to develop MCC is five-fold greater after organ transplantation and 11-fold greater for patients with AIDS compared to the general population [1]. Chronic arsenic exposure has also been implicated in the pathogenesis of MCC [4]. Although malignant melanoma is 50-fold more frequent than MCC, the prognosis for patients with MCC is worse [3]. The five-year survival rate for patients with a localized skin disease is 50–76%, 39% for patients with regional node disease, and 18% for patients with distant metastatic disease [2]. Clinical diagnosis of MCC is often difficult in the differential diagnosis since both basal cell carcinoma or squamous cell carcinoma are more common. MCC can also be mistaken for metastasis of neuroendocrine tumors, melanoma, or lymphoma [2]. Microscopic diagnosis can also be challenging. The typical presentations are uniform cells with round or oval nuclei that show expression of cytokeratin 20 (95%) and neuroendocrine markers, such as synaptophysin, chromogranin A, neuron-specific enolase, or CD56. Expression of p63 is found in 60% of MCC patients [2,5,6].

After the diagnosis of MCC, a complete excision (1–2cm margin) and adjuvant radiation is normally performed. Sentinel lymph node mapping and biopsy are recommended together with stage imaging (ultrasonography, computer tomography (CT), or positron emission tomography (PET)) [4]. Around 55% of patients with a distant metastatic MCC respond positively to first line treatment with chemotherapy consisting typically of platinum-based + etoposide. However, because the responses are not durable, tumors often recur by 4–15 months with a median progression-free survival of only 94 days and median overall survival of 9.5 months [3,7]. Although recent studies have shown some evidence of benefits in the long-term response for patients treated with the PD-L1 specific antibody avelumab [8], more effective novel therapeutic targets are needed.

Membrane transport proteins are involved in the movement of ions and small molecules across biological membranes. Many different membrane transporters have been shown to facilitate cancer development, progression, and drug resistance [9]. A recent study of Mycielska et al. showed that extracellular citrate is supplied to cancer cells through a plasma membrane-specific variant of the mitochondrial citrate transporter pmCiC, which is a member of the SLC25 mitochondrial transporter family [10]. This transporter enables cancer cells to use extracellular citrate for metabolism and supports cell proliferation. Citrate is a central metabolite for fatty acid synthesis, which is necessary for tumor growing. A lack of citrate also leads to morphological changes in cancer cells; thus, citrate seems to have a high impact on cancer growth [11]. Gluconate has been discovered as a specific and irreversible inhibitor for pmCiC [10,12,13], which opens the possibility for therapeutic intervention.

In this study, we have conducted experiments to explore the hypothesis that pmCiC is important for MCC tumors by first measuring expression in MCC cell lines and human tumor biopsies. We then tested the effects of extracellular citrate on MCC proliferation in vitro and applied gluconate-based pmCiC inhibition to these in vitro and in vivo systems to show whether the tumor promoting effects of citrate can be inhibited.

## 2. Results

### 2.1. Expression of PmCiC in Merkel Cell Carcinoma Cells and Merkel Cell Carcinoma Tissues

**Figure 1:**
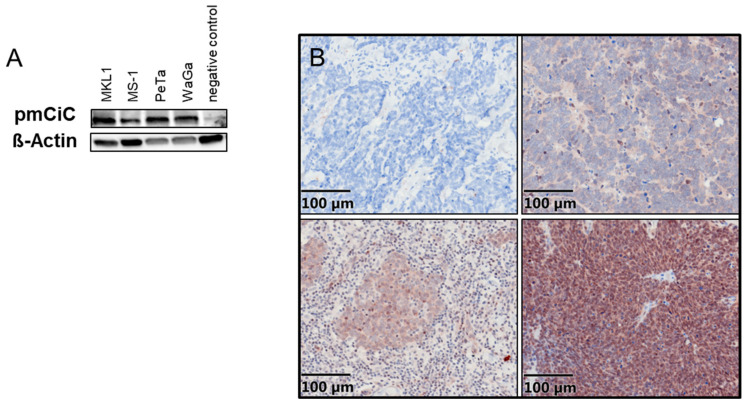
(**A**) Expression of pmCiC in 4 different cell lines (negative control: melanoma cells known for having no pmCiC; Figure S1). (**B**) PmCiC expression in Merkel cell carcinoma. Expression was ranked as negative (upper left panel), weak (upper right panel), intermediate (lower left panel), and high (lower right panel).

We first tested whether characterized MCC cell lines express pmCiC in vitro. All four MCC cell lines (WaGa, PeTa, MKL-1, MS-1) showed high expression of pmCiC by Western blotting (Figure 1A).

Next, we utilized immunohistochemical staining to detect pmCiC expression in formalin-fixed and paraffin-embedded primary MCC tissue sections. These results showed that 26 of the 28 samples stained positive for pmCiC (93%), where 2 samples showed no pmCiC expression (−), 9 showed weak/partial expression (+/−), 9 showed high expression (+), and 8 had very high expression (++) (Figure 1B). In total, 13 of the 28 patients had known metastatic disease, while 5 showed no metastases; clinical follow-up data were missing from the remaining 10 patients.

### 2.2. Proliferation of MCCs Is Increased in the Presence of Extracellular Citrate

**Figure 2:**
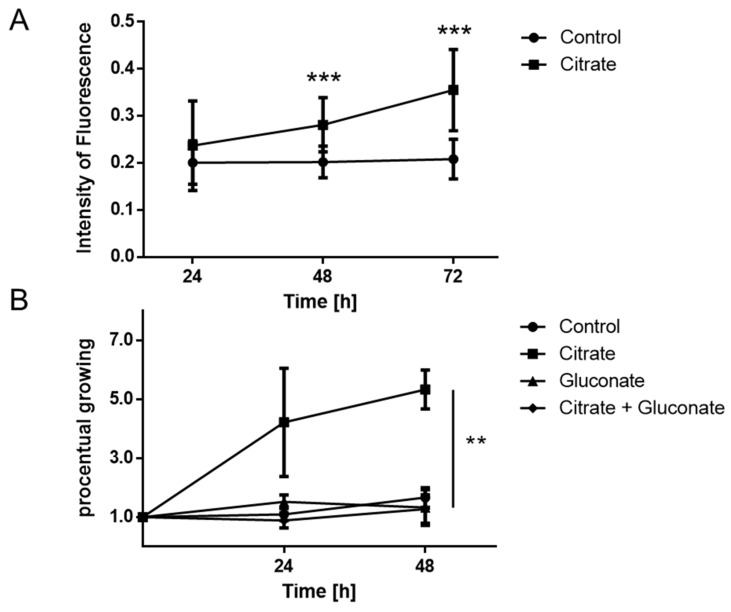
(**A**) Merkel cell carcinoma cells (MS-1) cultured with or without 200 µM citrate measured after 24 h, 48 h, and 72 h showing a significantly higher proliferation rate when treated with citrate (Method: CyQuant direct cell proliferation assay; *t*-test 48 h: *p* = 0.00036; 72 h: *p* = 0.00007). (**B**) Merkel cell carcinoma cells (PeTa) showed a higher number of cells after a treatment with 200 µM citrate. After adding 100 µM sodium gluconate, this effect was gone (Method: LUNA-FL™ Automated Fluorescence Cell Counter; *t*-test with citrate 48 h: control *p* = 0.0017; gluconate *p* = 0.0008; citrate + gluconate *p* = 0.0007; ** means *p* ≤ 0.01; *** means *p* ≤ 0.001).

To investigate the effect of citrate on MCC cells, we used a dialyzed serum that does not contain citrate in experimental cultures of MS-1 cells. For this experiment, one group of cultures was supplemented with 200 µM citrate and the proliferation rate in both groups was measured after 24, 48, and 72 h using the CyQUANT Cell Proliferation Assay. There was a significant difference at 48 and 72 h between cells treated with 200 µM Na+-citrate versus cultures without added citrate, (48 h: *p* = 0.00036; 72 h: *p* = 0.00007; Figure 2A). pH values were measured in these cultures, but no difference was observed in the presence or absence of added citrate (data not shown); therefore, the effect could not be attributed to the pH changes. Similar results were obtained for WaGa cells (data not shown). Another MCC cell line (PeTa) was exposed to similar conditions for 24 and 48 h, with and without added citrate, and cells were directly counted using the LUNA cell counter; direct cell counting was possible with this cell line, since they do not tend to form cell aggregates in culture, unlike the MS-1 cells. A significant difference in number of cells was found after 48 h of incubation with citrate, compared to the cells without citrate (Figure 2B, 12 technical and 3 biological replicates were performed; *t*-test with citrate 48 h: control *p* = 0.0017; gluconate *p* = 0.0008; citrate + gluconate *p* = 0.0007). After adding 100 µM Na^+^-gluconate to inhibit pmCiC-mediated citrate transport into cells, the proliferative effects of citrate were abrogated. Notably, Na^+^-gluconate treatment alone had no effect on PeTa MCC cell proliferation (Figure 2B). We also investigated whether a lack of citrate or treatment with Na^+^-gluconate might disguise cell proliferation effects via triggering apoptosis. Western blots for caspase 3, 7, 9, and PARP, as well as cleaved caspase 3, 7, 9, and cleaved PARP apoptosis markers did not reveal any evidence that the citrate or Na^+^-gluconate effects are related to apoptosis induction (data not shown).

### 2.3. Gluconate Inhibits Growth of Merkel Cell Carcinomas In Vivo

**Figure 3:**
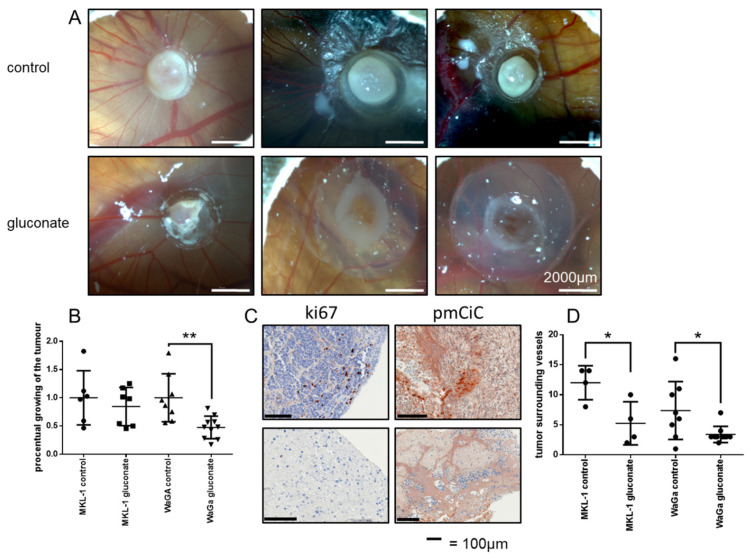
(**A**) Tumors in CAM-Assay after 5 days of treatment with sodium chloride (control) or sodium gluconate (gluconate; *n* = 20). (**B**) Diagrams after measurement of the tumor size showing a statistically significant result for WaGa (*t*-test MKL-1 *p* = 0.52; WaGa *p* = 0.01). (**C**) Ki67 and pmCiC expression in both groups. Ki67 is downregulated after a 5day treatment with sodium gluconate. (**D**) Significant differences in the number of tumor-surrounding vessels for WaGa and MKL-1 (*t*-test MKL-1 *p* = 0.03; WaGa *p* = 0.02; * means *p* ≤ 0.05; ** means *p* ≤ 0.01).

Bhat et al. showed that the Chick Chorioallantoic Membrane (CAM) model works well for in vivo testing of MCC tumor cell development [14]. In our experiments, the MCC cell lines WaGa and MKL-1 were established for use in this assay, with growing tumors showing high pmCiC expression by Western blotting (Figure 1A). Results from the CAM assay with MCCs WaGa showed significantly (*p* = 0.01) decreased tumor growth when treated with Na^+^-gluconate externally for 5 days compared to tumors treated with NaCl (Figure 3B). Na^+^-gluconate (concentration 500 mg/kg/d) or NaCl was applied every 24 h, as previously described (*n* = 20) [11]. Consistent with this observation, Ki67 expression was lower in tumors treated with Na^+^-gluconate (Figure 3C) and macroscopic findings showed smaller tumors with gluconate treatment. Immunohistochemistry staining of WaGa tumors showed high expression of the pmCiC, as expected. PmCiC expression was not decreased after gluconate treatment, suggesting that Na^+^-gluconate inhibits citrate transport but does not decrease its expression in these tumors. Together, these experiments show that blocking the pmCiC transport of citrate with Na^+^-gluconate in vivo results in a reduction in the growth of MCC tumors. By observing tumor-surrounding vessels, a lower number of vessels could be seen in tumors treated with sodium gluconate compared to the control group (Figure 3D). This effect was observed for MKL-1 (12 vs. 5.25 surrounding vessels, *p* = 0.03), as well as for WaGa (10 vs. 3.2 surrounding vessels, *p* = 0.02). These results suggest not only a direct effect of gluconate on proliferation, but also on angiogenesis.

## 3. Discussion

In our present study, we primarily provide evidence that citrate and pmCiC expression may play a role in the biology of human MCC. PmCiC is expressed in 93% of MCC tissue samples and in 4 out of 4 tested cell lines. In vitro, we could detect an effect of extracellular citrate on the proliferation rate of PeTa and MS-1 cell lines. This effect could be blocked in PeTa cells by adding Na^+^-gluconate as a specific pmCiC inhibitor. Growth inhibition was most probably due to cell cycle inhibition, rather than induction of apoptosis. We validated these findings using the 3D in vivo CAM model, where Na^+^-gluconate lowered MCC proliferation and showed antiangiogenic effects on tumor-surrounding vessels. Therefore, our study supports further research to therapeutically target the pmCiC in human MCC.

PmCiC expression in MCC human tissue was present in most of the specimens studied. Due to the incomplete clinical patient history available, it is difficult to make a firm conclusion about the potential correlation of staining intensity with disease outcome. However, our data strongly suggests that pmCiC is expressed in most patients with metastatic disease, which is consistent with our hypothesis that extracellular citrate uptake plays an important role in supporting metastatic behavior in vivo [10]. From a diagnostic perspective, since the pmCiC was essentially expressed in all aggressive tumor types studied until now, it will not necessarily be useful as a specific marker to facilitate MCC diagnosis; however, its potential to be used as a prognostic marker is worth exploring.

Citrate is the primary substrate in fatty acid synthesis, which is a metabolic hallmark of cancer [15]. By extension, citrate has also logically been considered a central metabolite in cancer cells [16]. Until recently, it was generally agreed that cancer cells synthesize citrate intracellularly mainly in the process of reverse carboxylation from glutamine [17], and use different metabolic pathways, including the serine pathway [18] and lactate metabolism [19], to secure sufficient amounts of the Krebs cycle intermediates. In this way, cancer cells are able to meet their needs for increased citrate synthesis under changing extracellular conditions, which allows for metabolic flexibility [20]. A recent study showed that besides intracellular synthesis of citrate, citrate is taken up extracellularly by epithelial-derived cancer cells, such as prostate, gastric, pancreatic, or liver [21]. Our present study shows that this is also the case for MCC cells. Since the pmCiC is expressed on many different types of cancer, we speculate that pmCiC-mediated uptake of extracellular citrate is a general mechanism of neoplastic cells to meet their metabolic need [11]. In vitro extracellular citrate supported tumor proliferation, but determining the exact mechanism requires further studies. Although in-depth analysis of human tumor pmCiC expression with correlation to metastasis and survival rates was not possible due to insufficient case numbers and an incomplete set of patient data, we plan to undertake these studies in the future.

Blocking of citrate uptake by cancer cells using Na^+^-gluconate as a specific pmCiC blocker [13] reduced subcutaneous tumor growth of human pancreatic L3.6pl cancer cells and changed the tumor’s metabolic characteristics [10]. The clearly smaller tumor (3D in vivo CAM-model) mass after Na^+^-gluconate treatment correlates with the lower MCC tumor cell proliferation rate when deprived of citrate [22]. Gluconate-treated tumors also showed evidence of reduced angiogenesis, which is critical for tumor growth and metastasis [23,24]. Mechanistic studies should be performed to determine if citrate or gluconate has a direct impact on the release of pro-angiogenic factors, such as IL-6, G-CSF, or PGF [25].

## 4. Materials and Methods

### 4.1. Immunohistochemistry

Paraffin-embedded sections were dewaxed using xylol (Merck, Darmstadt, Germany) and then rehydrated by washing twice with absolute ethanol, twice with 96% ethanol, and twice with 70% ethanol. After blocking endogenous peroxidases using 3% H_2_O_2_ for 10 min, samples were washed once with bi-distilled water, heated in HIER Citrate Buffer pH6 (Zytomed/Biozol, Eching, Germany) at 90 °C for 20 min, and cooled for 20 min. Samples were blocked using the blocking solution of the ZytoChem Plus HRP-kit Rabbit/Mouse (Zytomed) for 10 min.

Sections were labeled at 4 °C overnight using a rabbit monoclonal antibody against pmCiC (D2P2F, Cell Signaling; dilution for patient samples and for cell lines 1:200) and a rabbit monoclonal antibody against Ki67 (Abcam, Cambridge, UK; dilution for cell lines 1:1000). After washing with DPBS, sections were incubated with biotinylated secondary anti-rabbit (HRP060-RB) antibodies for 30 min, washed in DPBS, and incubated with streptavidin HRP conjugate for 20 min (all from ZytoChem Plus HRP Kit, Zytomed). After washing with DPBS, slides were stained using AEC+ High Sensitivity Substrate Chromogen Ready-to-Use (Dako/Agilent Technologies, Hamburg, Germany), counterstained using hematoxylin (Carl Roth, Karlsruhe, Germany), and mounted using Aquatex (Merck). Tissues were collected from patients of the department of dermatology at the university hospital of Regensburg and correlated with clinical outcome. Ethics permission No. 22-2834-104.

### 4.2. Cell Culture

Cell lines from primary Merkel cell carcinoma (PeTa), as well as from metastatic disease (WaGa, MS-1, and MKL-1), were grown in RPMI-1640 medium with 1% penicillin/streptomycin, 1% L-glutamine, and 10% fetal bovine serum. Although cell lines were not further authenticated, they were grown at low passage numbers from original sources and were kept typically in culture for only 2 months. Cells were tested and confirmed to be mycoplasma free. The following chemicals were used: citric acid and Na^+^- gluconate (Sigma, St. Louis, MO, USA), and dialyzed serum (PAN Biotech GmbH, Aidenbach, Germany). The following antibodies were used: pmCiC specific antibodies (12; custom-made by GenScript Inc., Piscataway, NJ, USA) and Ki67 (Abcam, Cambridge, UK). Experimental media consisted of glucose free RPMI-1640 (Lonza, Basel, Switzerland), 10% dialyzed serum, 2 mM glutamine, 0.25 g/L glucose, ±200 µM citrate, and ±100 µM Na^+^-gluconate, unless otherwise stated. The incubation time varied between 24 h, 48 h, 72 h, and 5 weeks, as specified. Western blots were analyzed by measurement of the pixel density using ImageJ software (Laboratory for Optical and Computational Instrumentation (LOCI), University of Wisconsin, Madison, WI, USA). The CyQUANT Direct Cell Proliferation Assay (Thermo Fisher Scientific, Waltham, MA, USA) was used to measure cell proliferation. Cells were counted with LUNA-FL™ Automated Fluorescence Cell Counter (Logos Biosystems, Anyang-si, Korea). Western blots were analyzed by measurement of the pixel density using ImageJ software.

### 4.3. Chick Chorioallantoic Membrane Model

The Chick Chorioallantoic Membrane (CAM) model was performed as described before [11,26,27]. Two days after implantation of 2 × 10^6^ Merkel cell carcinoma cells (WaGa and MKL-1), daily treatment with either Na^+^-gluconate (500 mg/kg/d) or NaCl 0.9% was performed (*n* = 20). Pictures were taken and tumors were removed after 5 days. The size of tumors was measured and the surrounding vessels were counted for all tumors.

### 4.4. Statistics

Statistics were performed using GraphPad by Dotmatics. A *t*-test was performed and significance was assumed for *p* < 0.05.

## 5. Conclusions

In conclusion, extracellular citrate uptake via the pmCiC is a potential novel therapeutic target in MCC tumors that highly expresses this critical citrate transporter, especially for cancers that tend to be more aggressive or metastatic. The exact mechanisms that play a role in this effect will require further investigation.

## Data Availability

The data presented in this study are available in this article.

## Supplementary Materials

The following supporting information can be downloaded at: https://www.mdpi.com/article/10.3390/cancers14143425/s1, Figure S1: Uncropped Western Blot Figures.
