# Supplementary figures and images for: The Role of Citrate Homeostasis in Merkel Cell Carcinoma Pathogenesis
Source: Cancers (Basel). 2022 Jul 14;14(14):3425. doi: 10.3390/cancers14143425 (PMC9325124; doi:10.3390/cancers14143425)

**Membran 2.1**

- **R-pmCic (old 1:200)**

Mild Stripping

- **M-Aktin C59**

**WB**

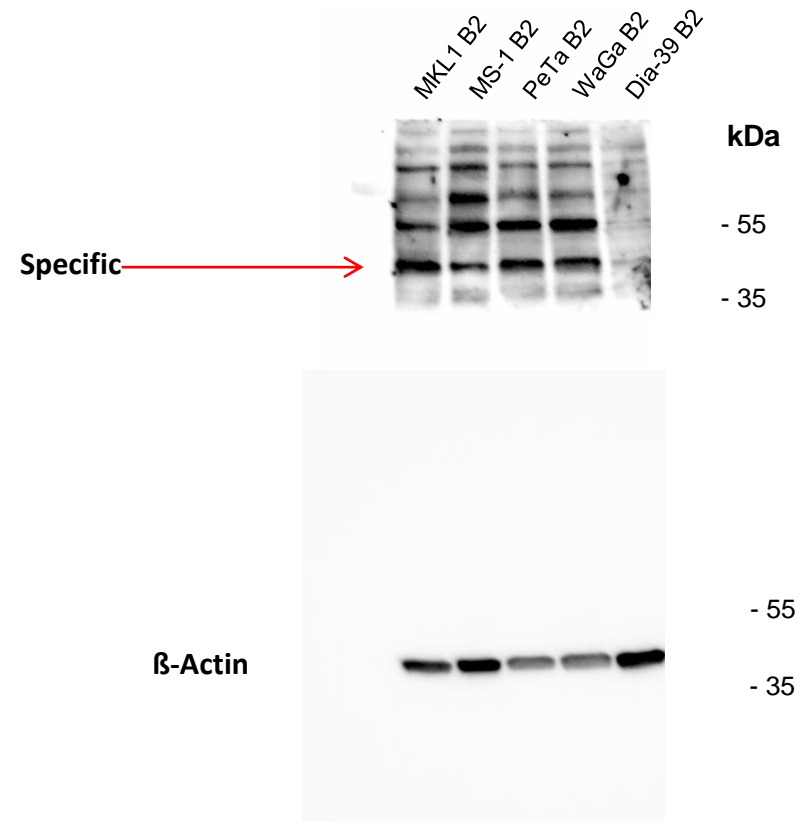

Supplement: Supplementary file 1 [file cancers-14-03425-s001.zip › cancers-1761447-supplementary.pdf]
